# 65 Exploring the experience of beach access in people living with a disability - a mixed methods study

**DOI:** 10.1093/eurpub/ckae114.064

**Published:** 2024-09-26

**Authors:** James Czencz, Georgia McKenzie

**Affiliations:** Timboon District Health Service, Timboon, Australia; St Vincent’s Hospital, Fitzroy, Australia

## Abstract

**Introduction:**

Access to blue spaces and opportunities to be active in the community are important to adults living with a disability. Beach access days are inclusive events that promote physical accessibility and social inclusion. This study aimed to explore the experiences of adults living with a disability attending a beach access day in South West Victoria.

**Methods:**

Employing a mixed-methods research design, five participants (Mean Age: 53.5 years, Male: 4) completed a validated beach accessibility survey tool comprising likert scale and short answer questions regarding barriers, facilitators, and experiences of beach access. Recruitment occurred following the beach access day, with survey links disseminated via email from registration data to eleven attendees. Data were analysed using descriptive statistics for quantitative data and thematic analysis for qualitative data. Participants were offered access to the preliminary study results for feedback.

**Results:**

Based on prelimanary findings, participating in a beach access day event positively impacted the attitudes of individuals with disabilities towards beach access. The qualitative data revealed three themes: 1. Socialisation and recreation - the need to socialise and engage in physical activity within the community; 2. Health and well-being - participants perceived improved accessibility as beneficial to their health; 3. Connection with identity - the beach is not just a place but a part of who they identify as. The quantitative survey data also identified barriers to beach access, including personal health issues, fatigue, difficulty moving on sand, and lack of transfer equipment. However, the study also found that certain facilitators, such as more accessible parking and toilets, transfer equipment like hoists, and physical assistance with beach activities, could help improve beach access for individuals with disabilities.

**Conclusion:**

The study highlights the need to make beaches accessible, and improving access can promote positive outcomes and the desire to participate more often. Inclusive events like beach access days have the potential to improve the health and well-being of people living with a disability and promote more inclusive and disability-aware societies. Communities should invest in such initiatives to make blue space environments more accessible and inclusive for individuals of all abilities.

